# Hard X-ray nanoprobe scanner

**DOI:** 10.1107/S2052252521007004

**Published:** 2021-07-31

**Authors:** Jumpei Yamada, Ichiro Inoue, Taito Osaka, Takato Inoue, Satoshi Matsuyama, Kazuto Yamauchi, Makina Yabashi

**Affiliations:** a RIKEN SPring-8 Center, 1-1-1 Kouto, Sayo, Hyogo 679-5148, Japan; b Division of Precision Engineering and Applied Physics, Graduate School of Engineering, Osaka University, 2-1 Yamada-oka, Suita, Osaka 565-0871, Japan; c Department of Materials Physics, Graduate School of Engineering, Nagoya University, Furo-cho, Chikusa, Nagoya 464-8603, Japan; d Japan Synchrotron Radiation Research Institute, 1-1-1 Kouto, Sayo, Hyogo 679-5148, Japan

**Keywords:** X-ray optics, X-ray nanoprobes, X-ray prisms, X-ray mirrors, scanning X-ray microscopy, hard X-rays

## Abstract

X-ray scientists are continually striving to improve the quality of X-ray microscopy, due to the fact that the information obtained from X-ray microscopy of materials can be complementary to that obtained from optical and electron microscopes. In contrast to the ease with which one can deflect electron beams, the relative difficulty to deflect X-ray has constrained the development of scanning X-ray microscopes (SXMs) based on a scan of an X-ray small probe. This restriction has caused severe complications that hinder progress toward achieving ultimate resolution. Here, a simple and innovative method for constructing an SXM equipped with a nanoprobe scanner is proposed. The nanoprobe scanner combines X-ray prisms and advanced Kirkpatrick–Baez focusing mirrors. By rotating the prisms on the order of degrees, X-ray probe scanning with single-nanometre accuracy can be easily achieved. The validity of the concept was verified by acquiring an SXM image of a test pattern at a photon energy of 10 keV, where 50 nm line-and-space structures were resolved. This method is readily applicable to an SXM with a single-nanometre resolution and will assist effective utilization of increasing brightness of fourth-generation synchrotron radiation sources.

## Abstract 2

A scheme for high-resolution X-ray microscopy by scanning the nanoprobe, rather than the sample, has been demonstrated. A combination of X-ray prisms and advanced reflective mirror optics offer a simple and precise nanoprobe scan that will open new opportunities for microscopic X-ray analyses.

## 1. Introduction   

The advent of bright synchrotron radiation (SR) sources has fostered the development of X-ray microscopy, which has provided structural, elemental and compositional information about specimens with a high spatial resolution (Guttmann *et al.*, 2012 ▸; Donnelly *et al.*, 2017 ▸; Shapiro *et al.*, 2014 ▸). A scanning X-ray microscope (SXM) is one of the principal X-ray microscopes (Horowitz & Howell, 1972 ▸; Rarback *et al.*, 1988 ▸). It illuminates a specimen with a tightly focused X-ray probe to acquire an image by scanning a relative position between the probe and the specimen. The spatial resolution of an SXM is fundamentally determined by the focusing spot size. As a practical matter, the resolution in an SXM is effectively limited by the scanning accuracy. Recent progress in SR sources and X-ray nanofocusing optics has drastically reduced the probe size to a single-nanometre scale (Yamauchi *et al.*, 2011 ▸; Mohacsi *et al.*, 2017 ▸; Bajt *et al.*, 2018 ▸). The performance of SXMs eventually became limited by the scanning systems, not the size of the X-ray probe.

In a scanning electron microscope that has reached outstanding resolution (Peter & John, 2019 ▸), the probe is precisely steered by electromagnetic deflectors. Although similar steering of the X-ray probe looks straightforward in an SXM, X-ray steering has hardly ever been adopted due to the lack of an appropriate X-ray optical device. For instance, a diffractive grating could be used to deflect the X-ray while the diffraction efficiency for a specific order is low. A total-reflection mirror could also be used but the nanometre-level shape accuracy and the nanoradian-level control that are needed to steer the X-ray nanoprobe are technically difficult to achieve. Consequently, mechanical scanning instruments for the specimens, not for the X-ray probe, have been pursued in scanning X-ray microscopy for half of a century. High-precision scanning systems with an accuracy of ∼20 nm have recently been constructed (Kilcoyne *et al.*, 2003 ▸; Nazaretski *et al.*, 2015 ▸; Villar *et al.*, 2018 ▸), while intricate instruments, such as positioning stages with closed-loop feedback using additional metrology, heat-rejected mechanics and huge granite bases, have complicated the realization of further accuracy and constrained the specimen environments. Some approaches of translating the light-weight focusing optics have been implemented for relatively coarse probe scanning (Arndt & Scotcher, 2002 ▸; Deng *et al.*, 2019 ▸). However, the same difficulties in the mechanical instruments have limited the achievable scanning accuracy. Although fourth-generation SR sources with increasing brightness can drastically enhance the SXM performance (Eriksson *et al.*, 2014 ▸; de Jonge *et al.*, 2014 ▸; Raimondi, 2016 ▸), widespread applications of ultimate-resolution SXMs with diverse specimens have been impeded by specimen scanning restrictions.

**Figure 1:**
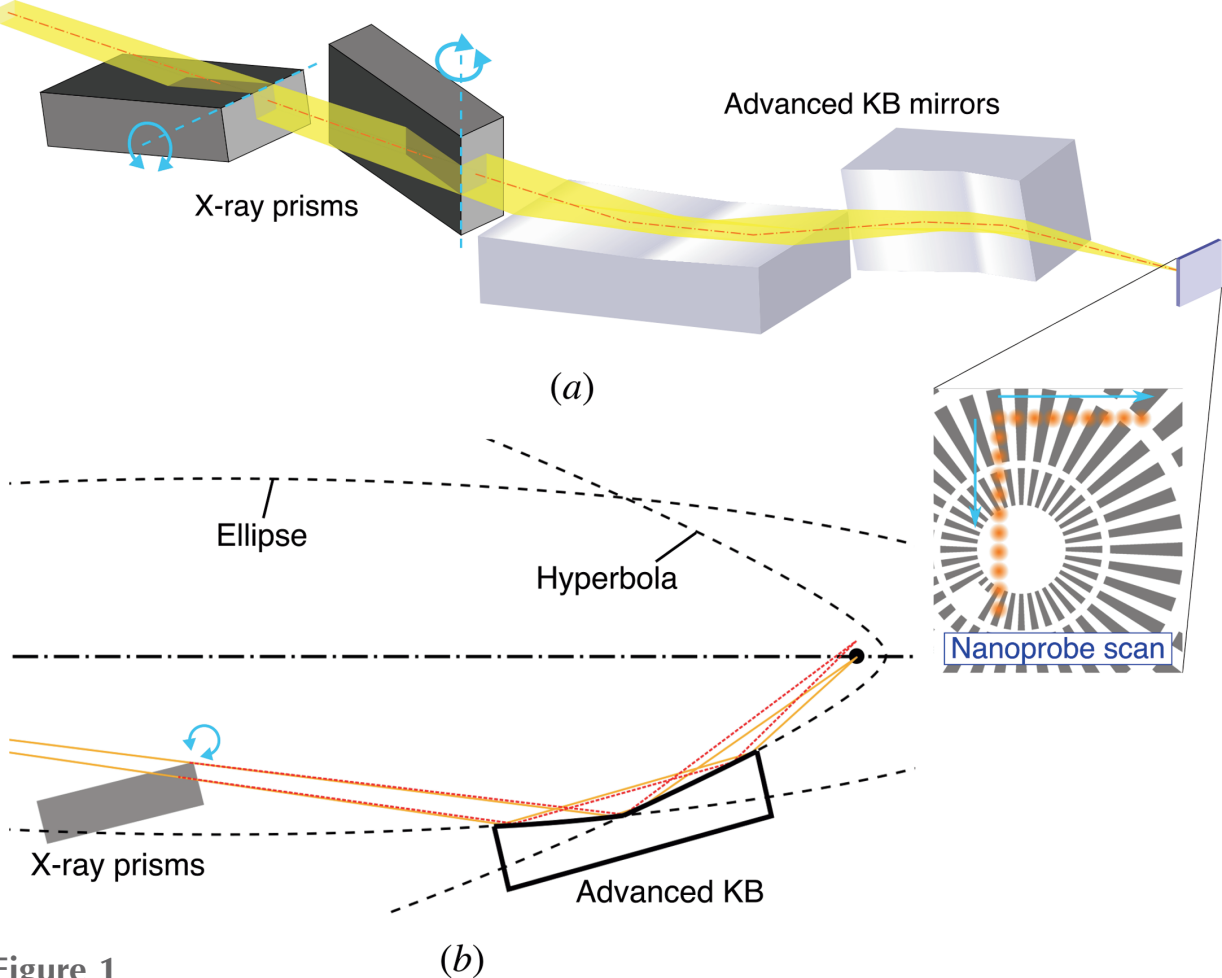
A conceptual schematic of the hard X-ray nanoprobe scanner. (*a*) A schematic of the hard X-ray nanoprobe scanner. (*b*) A cross section of the scheme.

In this article, we propose a scheme to steer an X-ray nanoprobe for an ultrahigh-resolution SXM. The optical components consist of X-ray prisms (Lang & Makepeace, 1999 ▸; Cederström *et al.*, 2000 ▸; Inoue *et al.*, 2018 ▸) used to deflect the incident X-ray beams and advanced Kirkpatrick–Baez (AKB) mirrors (Matsuyama *et al.*, 2017 ▸) used to generate a nanoprobe [see Fig. 1 ▸(*a*)]. The working principle is described as follows: the prisms are rotated to steer the X-ray beam through the prisms. Because the deflection response to the change in the rotation angle is quite small, the deflection angle can be precisely controlled with nanoradian accuracy by rotations of the prisms by angles in the order of a degree (∼10^−2^ radians). Then, the X-ray is reflected and focused by the AKB mirrors, which consist of two pairs of elliptical and hyperbolic mirrors, *i.e.* four-bounce reflection optics, as shown in Fig. 1 ▸(*b*). Thanks to the imaging capabilities of the AKB mirrors that can satisfy Abbe’s sine condition (Born & Wolf, 2001 ▸), the angular deflection of the incident beam to the AKB mirrors results in spatial translation of the nanoprobe on the focal plane without degradation of the focus size. This characteristic contrasts with that of conventional KB mirrors consisting of only elliptical mirrors (Kirkpatrick & Baez, 1948 ▸), where crucial off-axis coma aberration is generated. The total-reflection focusing mirror can generate an intense X-ray nanoprobe owing to its large spatial acceptance and high reflectivity, compared with focusing zone plates (Odstrcil *et al.*, 2019 ▸; Deng *et al.*, 2019 ▸).

## 2. Results   

### 2.1. Performance of X-ray prisms   

**Figure 2:**
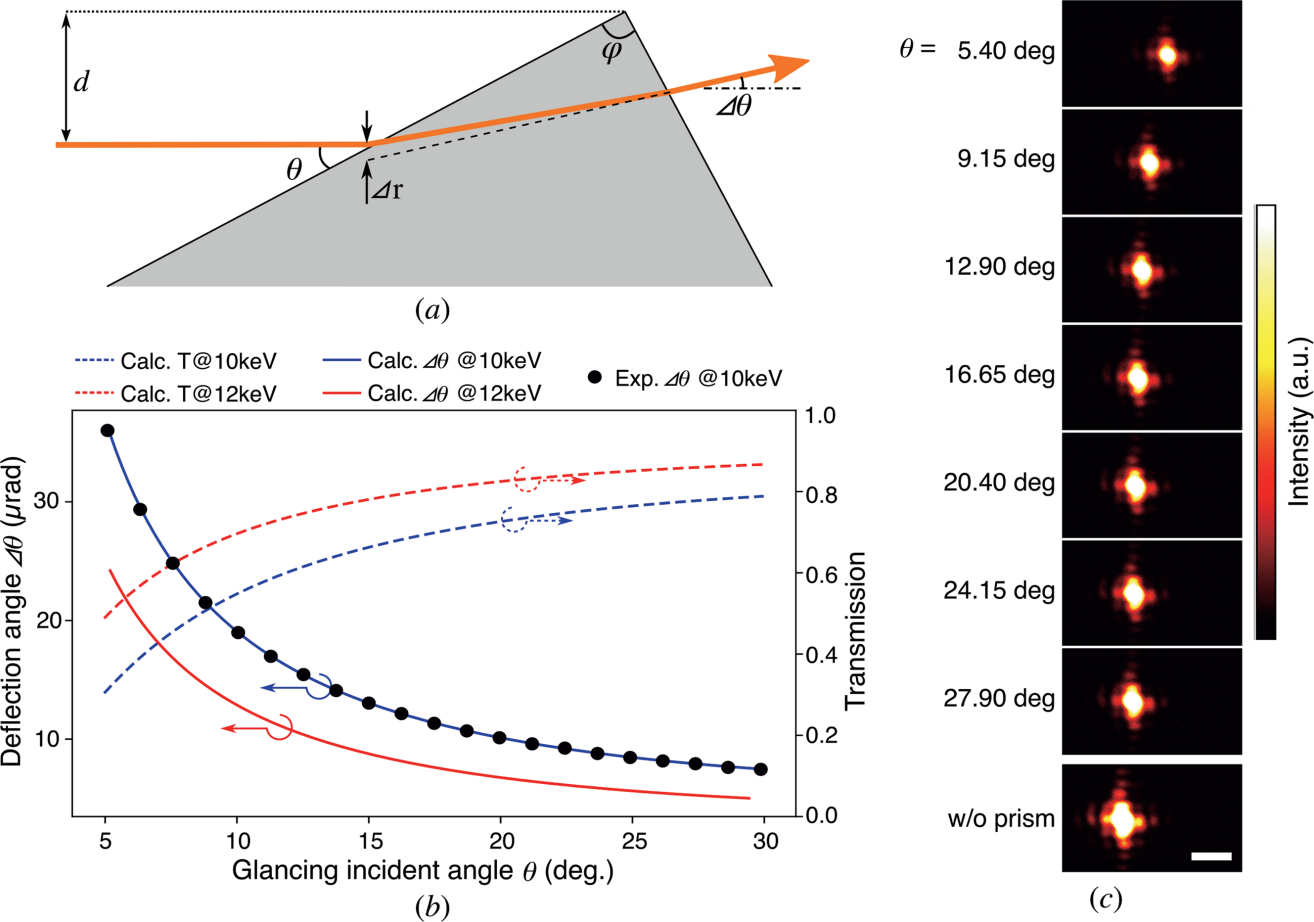
Schematic and performance of an X-ray prism. (*a*) A schematic of an X-ray prism and the trajectory of a deflected X-ray beam. (*b*) Incident-angle dependence of the deflection angle and transmission of the prism. The solid blue (red) line indicates the calculated deflection angle at a photon energy of 10 keV (12 keV). The dashed blue (red) line indicates the calculated transmission at 10 keV (12 keV), when the incident-beam width is 0.6 mm (see the supporting information). The black dots indicate the deflection angles examined by the experiment. (*c*) Images of the deflected X-ray beams while the incident angle to the prism was varied from 5.4 to 27.9°. The bottom image shows the result without the prism. The scale bar denotes 30 µm.

The deflection angle of the X-ray prisms Δθ is approximately given by (Inoue *et al.*, 2018 ▸)
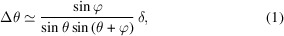
where δ denotes the phase-shifting part of the refractive index, and θ and φ denote a glancing incident angle and a prism apex angle, respectively, as illustrated in Fig. 2 ▸(*a*). Assuming a prism apex angle φ of 90°, one can simplify equation (1) as
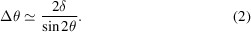
In Fig. 2 ▸(*b*), the Δθ versus θ relationships are shown as solid lines, which were calculated with the following parameters: a φ of 90°, a prism material of glassy carbon (density ρ = 1.51 g cm^−3^) and photon energies of 10 and 12 keV (δ = 3.138 × 10^−6^ and 2.178 × 10^−6^, respectively). Notably, Δθ is in units of µrad, whereas θ is in degrees. The ratio of change in *θ* to that in Δθ is 1:2.9 × 10^3^–1.2 × 10^5^, which leads to high-accuracy scanning by the prism rotational scan. The relative shift of the ray Δ*r* in the deflection is negligibly small, *e.g.* ∼3 nm or less, when the distance between the incident ray and apex *d* is less than 1 mm (see the supporting information). A small glancing incident angle elongates the path in the prism, which increases X-ray absorption. Nevertheless, the transmittance is acceptable for the SXM when one chooses a high photon energy above ∼10 keV and low-atomic weight materials for the prisms [see Fig. 2 ▸(*b*) dashed lines and the supporting information].

A nanoprobe scan experiment was performed at SPring-8 BL29XU (Tamasaku *et al.*, 2001 ▸) with a photon energy of 10 keV. X-ray prisms with apex angles of 90° made of glassy carbon (Tokai Carbon Co. Ltd) were utilized. The incident and exit surfaces of the prisms were polished. At first, deflection behavior was tested. An X-ray beam from a slit with a 15 µm square aperture irradiated the prism arranged in the horizontal direction. The deflected beam was monitored by a high-resolution X-ray camera [see *Methods* [Sec sec4] and Kameshima *et al.* (2019 ▸)], which was placed 0.988 m downstream from the prism. The incident angle was varied by rotating the prism, whose rotation center was at the apex. The deflection angles were determined using the camera length and displacements of the beam calculated by the cross correlation between images of the deflected beams and an image of the beam without the prism [Fig. 2 ▸(*c*)]. The obtained relationship between the deflection angle and the glancing incident angle was in quite good agreement with the calculation, as shown via the black dots in Fig. 2 ▸(*b*). Based on the results, prism scan functions for linearly steering the deflection angle were calculated with a simple fitting of equation (2). The fitted accuracy was 17 nrad for a root mean square (r.m.s.), including the uncertainty of the cross correlation. This result guaranteed a scanning accuracy of at least 2.9 nm r.m.s. on the focal plane.

### 2.2. Nanoprobe characterization   

**Figure 3:**
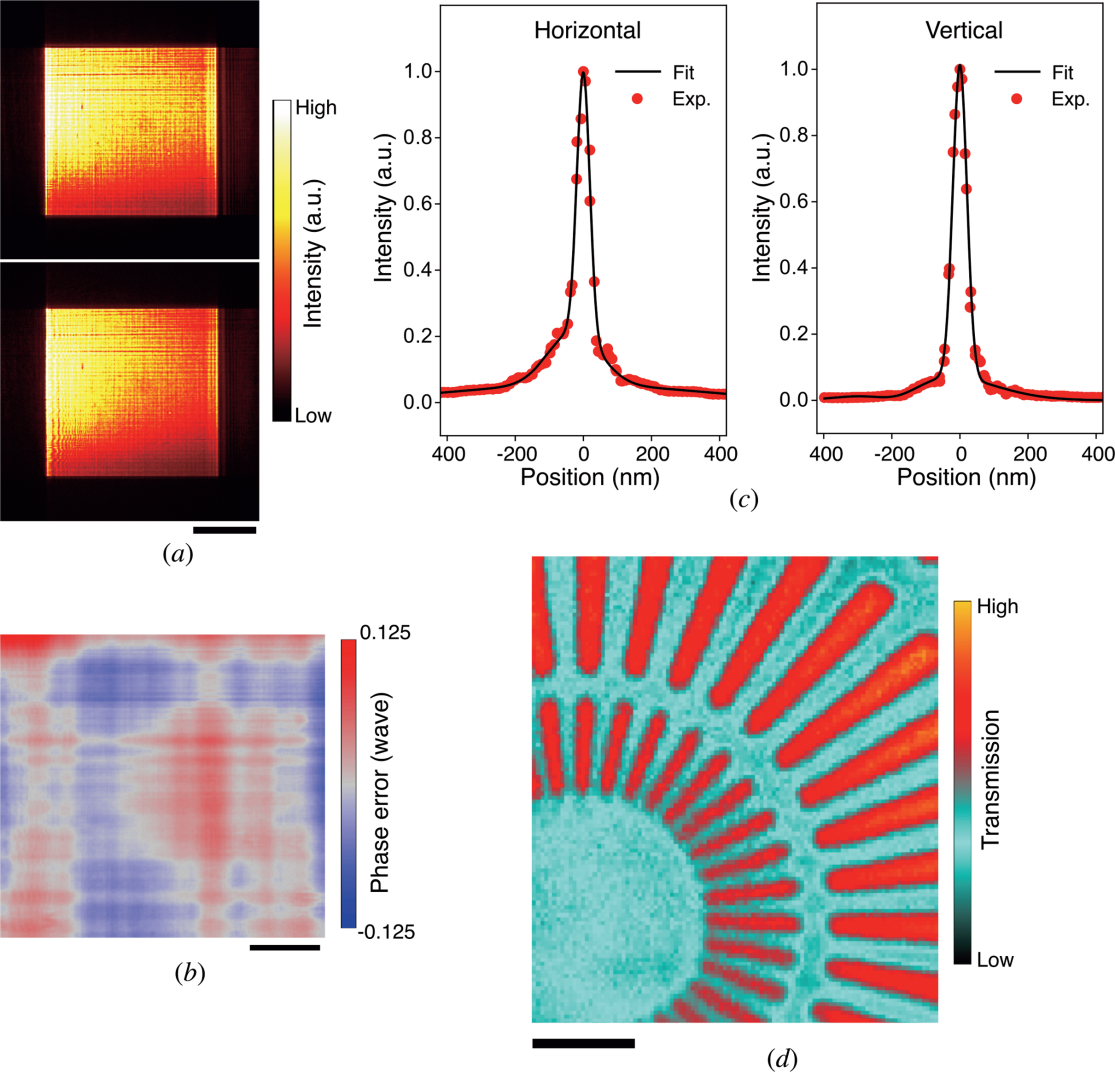
Demonstration results of the nanoprobe scanner. (*a*) Far-field images of the focused X-ray without (top) and with (bottom) the prisms. The scale bar denotes 1 mm. (*b*) The two-dimensional wavefront error of the nanoprobe scanner at the grating plane. The scale bar denotes 50 µm. (*c*) Focusing profiles in the horizontal (left) and vertical (right) directions. The red dots indicate the experimental data. The black solid lines represent the fitting results with the sum of three Gaussian functions. The focus size was 53.2 nm (H) × 52.5 nm (V) in FWHM. (*d*) An SXM image obtained using the nanoprobe scanner. A transmission image of the radial test pattern made of 500 nm thick tantalum. The scale bar denotes 0.5 µm.

Next, a vertical prism and the AKB focusing mirrors were added and aligned. The mirrors were developed in a previous study (Matsuyama *et al.*, 2017 ▸) and achieved nearly diffraction-limited performance. The reflected X-ray far-field images with and without the prisms are shown in Fig. 3 ▸(*a*). The images were obtained by using a large field-of-view X-ray camera (see *Methods* [Sec sec4]), which was placed 0.999 m downstream from the focus. Characteristic stripes in both images originated from the remaining high-spatial-frequency shape errors on the mirrors (Hu *et al.*, 2021 ▸). Although the absorption by the prisms created additional gradients in intensity across illumination apertures, speckle-like distortions did not appear, which indicates that the density of the prisms is sufficiently uniform. Additionally, the wavefront aberration, including fidelities of the prisms and the AKB mirrors, was measured by single-grating interferometry (Yamada *et al.*, 2020 ▸). Fig. 3 ▸(*b*) presents the obtained two-dimensional wavefront aberration after subtracting the second-order function including terms accounting for defocus and astigmatism. The peak-to-valley (PV) value of the 0.22 wave and the r.m.s. value of the 0.025 wave were obtained and are less than Rayleigh’s rule (*i.e.* 1/4 wave PV) and Maréchal’s criterion (*i.e.* 1/14 wave r.m.s.), respectively (Born & Wolf, 2001 ▸), which indicates that the prisms hardly deteriorated the wavefront. According to the results of knife-edge-scan measurements with 50 µm diameter gold wires, the focus size was measured to be 52.5 [vertical (V)] × 53.2 [horizontal (H)] nm in full width at half-maximum (FWHM) [see Fig. 3 ▸(*c*)].

### 2.3. SXM image using nanoprobe scanner   

After the nanoprobe characterization, an SXM image was obtained using the nanoprobe scanner. X-ray transmission through a radial test pattern (XRESO-50HC, NTT Advanced Technology Co.) was acquired using two intensity monitors placed just upstream from the mirrors and downstream from the pattern. The result is shown in Fig. 3 ▸(*d*). A clear image, in which inner-most structures were 50 nm lines and spaces, was obtained with a pixel size of 19.8 nm, 100 × 133 pixels and a total measurement time of ∼95 min. The high-resolution image without notable distortion represents the accuracy and validity of the proposed scheme.

## 3. Discussion   

In the SXM measurement, the prism was rotated with 0.8–27 mrad step to scan the probe translation with 19.8 nm step. Because the mechanical rotation stages have enough accuracy (∼4 µrad), the single-nanometre resolution is readily achievable by applying currently developed sub-10 nm focusing AKB mirrors (Yamada *et al.*, 2019 ▸; Inoue *et al.*, 2020 ▸). In this proof-of-principle study, the nominal speed of the scan was limited to 0.43 s pixel^−1^. A modification to on-the-fly spin scanning of asymmetric cone-shaped prisms (see the supporting information) will enable a pixel-transit time of a few tens of microseconds, leading to an image-measurement time of 1–20 s for the high-flux fourth-generation SR sources (de Jonge *et al.*, 2014 ▸). There is still some room to improve the performance of the nanoprobe scanner. Adaptable candidates for the improvement are diamond/Be/Si/Al prism materials depending on the photon energy, multi-deflection (compound) configurations, the Risley prism arrangement, *etc*. For the optimization of the AKB mirrors, a design involving long focal lengths can be used to extend the field of view; alternatively, short focal lengths can be used to increase the scanning accuracy. The scheme is also applicable to X-ray ptychography which is a diffraction-type scanning X-ray microscopy with an iterative phase reconstruction (Rodenburg *et al.*, 2007 ▸; Thibault *et al.*, 2008 ▸). Even though slight variations in the probe function may perturb the reconstruction, the normalization of the diffraction patterns by the incident intensities (Schropp *et al.*, 2013 ▸) and/or the probe relaxation-enhanced algorithms (Thibault & Menzel, 2013 ▸; Odstrcil *et al.*, 2016 ▸) can be used to manage such variations.

The hard X-ray nanoprobe scanner demonstrated here is based on an entirely simple principle and can readily achieve single-nanometre accuracy that is nearly free from scanning errors and thermal instability of the mechanical stages. The nanoprobe-scanning concept will certainly enable new opportunities for microscopic X-ray analyses of objects that are scarcely suited for precise scanning, *e.g.* materials fixed in massive instruments and fragile biological tissues with cryogenic apparatuses. The scheme will pave the way towards an ultimate-resolution SXM for various scientific investigations, especially in the fourth-generation SR sources.

## 4. Methods   

### 4.1. Experiments   

Scintillator-based X-ray cameras were utilized for the deflection-angle measurements and the wavefront characterization. A high-resolution camera for the deflection-angle measurements, consisting of a LuAG:Ce scintillator (Kameshima *et al.*, 2019 ▸), microscopic lenses, mirrors for visible light and a scientific complementary metal-oxide semiconductor detector (ORCA-Flash4.0 V2, Hamamatsu Photonics K.K.), had effective pixel sizes of 325 nm and 2048 × 2048 pixels. A large field-of-view camera for the wavefront characterization, which was a combination of Hamamatsu Photonics K.K. products (AA40 and ORCA-Flash4.0 V2), had effective pixel sizes of 3 µm and 2048 × 2048 pixels. The prisms had dimensions of 40 [length (*L*)] × 10 [width (*W*)] × 2 [thickness (*T*)] mm. The vertical (horizontal) prism was placed 0.41 m (0.27 m) upstream from the upstream edge of the AKB focusing mirror. The rotation centers of the prism scanners were tuned to coincide with the apexes with several-micrometre accuracy using a visible-light microscope. In BL29XU, the X-ray from an undulator was monochromated by Si(111) double crystals. For nanofocusing with AKB mirrors, the secondary source (SS) slit was set to a 30 µm aperture for the horizontal direction and a 10 µm aperture for the vertical direction. The SS slit was placed ∼45 m upstream from the mirrors. During the wavefront measurement and the knife-edge scan, the prisms were set to glancing incident angles of 9.1° (H) and 14.2° (V). The SXM image was obtained using a step scan of the prisms and scanning ranges of the glancing incident angles of 5.534–30.838° (H) and 9.648–33.846° (V).

### 4.2. AKB mirrors   

The AKB mirrors used in this study were designed with the following parameters: maximum glancing incident angles of 5.5 mrad, a numerical aperture of 1.5 × 10^−3^, substrate lengths of 80.0 mm (H) and 232.2 mm (V), a source–mirror distance of ∼45 m, focal lengths of 70.7 mm (H) and 229.6 mm (V), a working distance of 33 mm, and magnification factors of 637 (H) and 196 (V). The angles of view for the AKB mirrors were estimated to be ±80 µrad (H) and ±50 (V) µrad, which are larger than the angular scanning range for the prism deflection in this experiment. As a side note, the angle of view of a conventional KB mirror with similar size, arrangement and focal length is ±0.5–1.4 µrad, which indicates the advantage of the AKB mirrors for the nanoprobe-scanner application. Pairs of elliptical and hyperbolic shapes were fabricated in single synthetic silica substrates, as described by Matsuyama *et al.* (2015 ▸). The mirror surfaces were coated with chromium binding layers and platinum 100 nm thick layers.

### 4.3. Single-grating interferometry   

The single-grating interferometer consisted of one-dimensional π/4 phase gratings and a large field-of-view type X-ray camera. The gratings, which were made of tantalum with periods of 2.5 µm (NTT Advanced Technology Co.), were arranged 82.4 mm downstream from the focus. The horizontal and vertical self-images with Talbot orders of 1.5 were acquired individually, and the differential phases were analyzed by the Fourier transform method (Takeda *et al.*, 1982 ▸). The two-dimensional wavefront was calculated through the cosine-transform integration method (Huang *et al.*, 2015 ▸).

## 5. Related literature   

The following references are cited in the supporting information for this article: Als-Nielsen & McMorrow (2011 ▸).

## Supplementary Material

Supporting information. DOI: 10.1107/S2052252521007004/ro5029sup1.pdf
