# Supplementary material for: Hard X-ray nanoprobe scanner
Source: IUCrJ. 2021 Jul 31;8(Pt 5):713–8. doi: 10.1107/S2052252521007004 (PMC8420768; doi:10.1107/S2052252521007004)
Supplement: Supplementary file 1 [file m-08-00713-sup1.pdf]

IUCrJ

Volume 8 (2021)

Supporting information for article:

Hard X-ray nanoprobe scanner

Jumpei Yamada, Ichiro Inoue, Taito Osaka, Takato Inoue, Satoshi Matsuyama, Kazuto Yamauchi and Makina Yabashi

The deflection in the X-ray prism is expressed in detail as follows. The refraction trajectory angles for prisms $\theta, \theta_1, \theta_2, \theta_3$ are defined in Figure S1. When the X-ray beams enter the prism surface with a glancing incident angle θ, from Snell's law, the glancing refraction angle θ_1 is given by

$$\theta_1 = \arccos(\frac{\cos\theta}{1-\delta}),$$

where δ denotes the phase-shifting part of the refractive index. Additionally, the refraction angles at the exit surface can be written as

$$\theta_3 = \arccos(\frac{\cos\theta_2}{1-\delta}).$$

The θ_2 can be simply written as

$$\theta_2 = \pi - \varphi - \theta_{1.}$$

From these angles, the deflection angle $\Delta\theta$ can be calculated using the relationship $\Delta\theta = \theta - \theta_1 - \theta_2 + \theta_3$. Assuming the small angle approximation of $\theta - \theta_1 \ll 1$ and $\theta_3 - \theta_2 \ll 1$, Equation (1) in the main text can be derived.

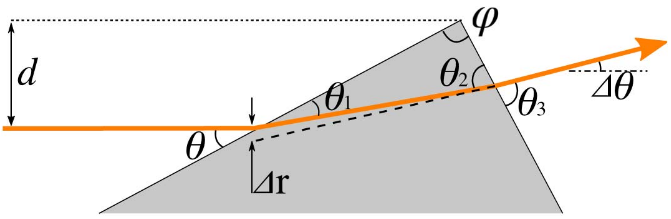

Figure S1 Schematic illustration of an X-ray prism and trajectory of the X-ray beam.

The relative shift Δr of the deflected ray, illustrated in Figure S1, is minimized when θ_2 is 90° (corresponding to $\varphi = \pi/2 - \theta_1$). The prism rotation scan will vary θ_2, and Δr cannot be exactly 0 through the scan, but it can be certainly compensated by modifying the rotational scan function. In this study, Δr was ~ 2 nm and ignored. Moreover, a divergent angle and monochromaticity of the incident beam cause prism aberration. However, general synchrotron X-ray beamlines using undulator sources and Si(111) double-crystal monochromators can generate hard X-ray beams with divergent angles of less than several tens of μrad and a monochromaticity $\Delta E/E$ of less than 5×10^{-4}. In this case, their average dispersion is ~8 nrad of the deflection angle, corresponding to a ~0.5 nm broadening on the focal plane in the proposed scheme, which is negligible.

When the X-ray beam is separated by a distance d from the straight line penetrating the apex of the prism, the path length in prism l can be written as

$$l = \frac{d}{\sin\theta}\{\cos\theta_1 - \frac{\sin\theta_1}{\tan(\theta_1 + \varphi)}\}.$$

Then, the transmittance is given by exp(-μl) where μ denotes the linear absorption coefficient [1]. Assuming a flat-top incident beam with width w where one edge of the beam irradiates the apex of the prism, the average transmittance T_{ave} can be written as

$$T_{\text{ave}} = \exp[-\frac{\mu}{w}\int_0^w \frac{x}{\sin\theta}\{\cos\theta_1 - \frac{\sin\theta_1}{\tan(\theta_1 + \varphi)}\}dx].$$

The photon energy and incident angle dependences of T_{ave} and $\Delta\theta$ were calculated using a w of 0.6 mm, φ of 90°, and the prism material parameters of glassy carbon. The results are shown in Figure S2(a). Comparing the maps of T_{ave} and $\Delta\theta$, a similar trend can be seen, *i.e.*, the transmission (deflection angle) is proportional (inversely proportional) to both the photon energy and glancing angle. This is the reason for the trade-off relationship between the angular scanning range and transmittance of the X-ray prisms. For practical application of the nanobeam scanner, considerably low transmission should be avoided. Assuming transmission thresholds of 0.35, 0.5 and 0.65, achievable angular scanning ranges were calculated, as shown in Figure S2(b). A large practical scanning range can be attained in the photon energy range higher than 10 keV, and hence, the proposed nanobeam scanner is suitable for hard X-rays. As a side note, the apex angle φ is hardly critical to the performance. As shown with the dashed blue line in Figure S2(b), the scanning range with a φ of 45° is almost the same as that with 90°.

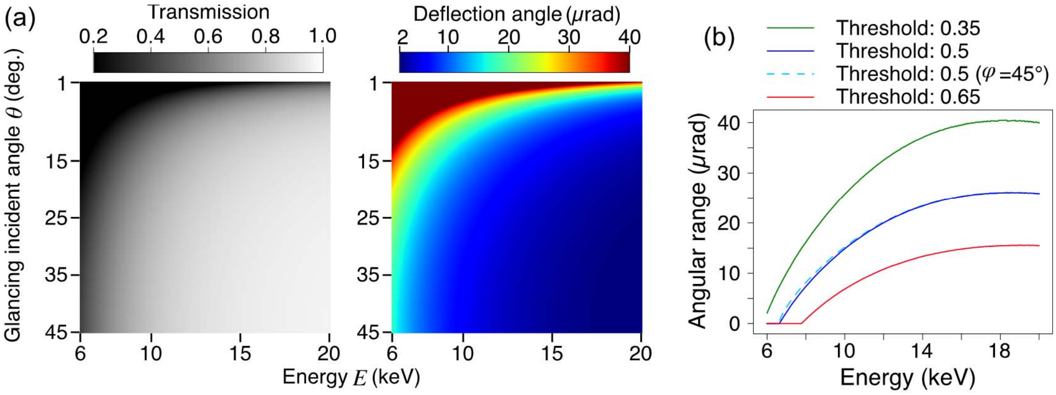

Figure S2 Transmission and deflection angle of the X-ray prism. (a) Energy and incident angle dependence of the transmission (left) and the deflection angle (right). (b) Angular range of the deflection

when the acceptable transmission is defined as 35 % (green), 50 % (blue), and 65 % (red). The blue dashed line on the right denotes the result for a prism apex angle of 45° and threshold of 50 %.

To improve the scanning speed, modification of the prism shape was considered. Figure S3 shows the asymmetric-cone prism candidate referenced in the main text. This shape enables steering of the X-ray beam with a continuous-spin motion. It has been pointed out that the competition for the fast SXM construction will face the scanning vibration problem: the scanning with a pixel transit time of ~15 μs would shake the specimen at ~100 Hz [2] in the specimen-scanning instruments. Spin scanning of the asymmetric-cone prism combined with the nanoprobe scanner, however, will be a preferable option for fast SXM.

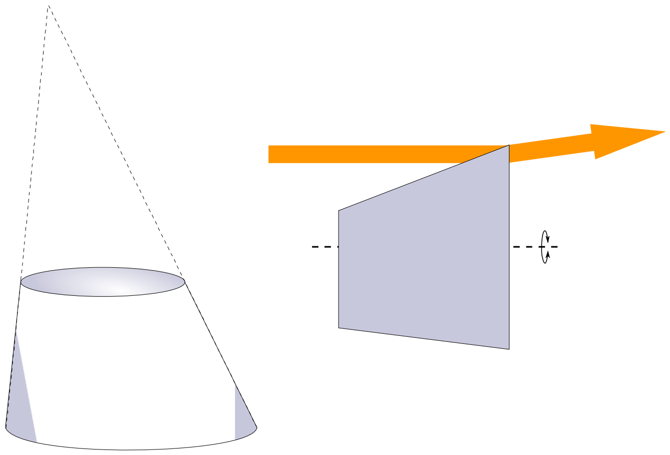

Figure S3 Schematic of the asymmetric-cone prism for a fast scanning speed.

References

[1] Als-Nielsen, J. & McMorrow, D. *Elements of Modern X-ray Physics, Second Edition.* (John Wiley & Sons, Ltd., New Jersey, 2011).

[2] de Jonge, M. D., Ryan, C. G. & Jacobsen, C. J. X-ray nanoprobes and diffraction-limited storage rings: opportunities and challenges of fluorescence tomography of biological specimens. *J. Synchrotron Rad.* **21**, 1031-1047 (2014).
